# Can Insulin Drops Accelerate Corneal Healing After Corneal Cross-Linking? A Preliminary Case Series †

**DOI:** 10.3390/medicina61122101

**Published:** 2025-11-26

**Authors:** Freja Bagatin, Ante Vukojević, Karla Ranđelović, Ivana Radman, Renata Iveković, Valentina Lacmanović Lončar, Ivanka Petric Vicković, Zoran Vatavuk

**Affiliations:** Department of Ophthalmology, Sestre Milosrdnice University Hospital Centre, 10000 Zagreb, Croatia; ante.vukojevic@kbcsm.hr (A.V.); karla.randelovic@kbcsm.hr (K.R.); ivana.radman@kbcsm.hr (I.R.); renata.ivekovic@kbcsm.hr (R.I.); valentina.lacmanovic.loncar@kbcsm.hr (V.L.L.); ivanka.petric.vickovic@kbcsm.hr (I.P.V.); zoran.vatavuk@kbcsm.hr (Z.V.)

**Keywords:** keratoconus, corneal cross-linking (CXL), corneal epithelial healing, topical insulin, epithelial defect

## Abstract

*Background and Objectives:* Corneal cross-linking (CXL) is the standard treatment for progressive keratoconus, but delayed epithelial healing remains a concern, increasing infection risk and patient discomfort. Studies suggest that insulin may promote corneal epithelial cell migration and proliferation, potentially accelerating wound healing. Its benefit has been observed in neurotrophic keratitis and diabetic epithelial defects, and it may offer similar effects post-CXL. Our objective is to evaluate the effect of topical insulin on epithelial healing after CXL in a small case series. *Materials and Methods:* Eight patients undergoing CXL for keratoconus were divided into two groups (*n* = 4 each). The insulin group received topical insulin eye drops (1 IU/mL in Systane^®^) five times daily, in addition to standard postoperative care. The control group received Systane^®^ alone with the same regimen. Daily follow-up included slit-lamp exam, anterior segment OCT, and photodocumentation until epithelial defect closure. *Results:* Baseline parameters (central corneal thickness, keratoconus stage, Schirmer test, tear break up test) were comparable. While not statistically significant, the insulin group showed numerically smaller epithelial defects on day 2, suggesting a possible trend toward faster healing. By day 3, re-epithelialization was complete in all patients. Pain decreased over time in both groups without significant differences. No adverse effects were noted. *Conclusions:* Topical insulin may modestly accelerate epithelial healing after CXL, as suggested by smaller defects on day 2 in the insulin group. Although results were not statistically significant, the trend warrants further investigation in larger studies.

## 1. Introduction

Keratoconus is a progressive, degenerative disorder of the cornea characterized by stromal thinning and conical protrusion, leading to irregular astigmatism and impaired visual acuity. The condition can significantly reduce the quality of life, particularly in young patients [1]. Corneal collagen cross-linking (CXL) is currently the only evidence-based treatment capable of halting or slowing the progression of keratoconus and other corneal ectasias by increasing the biomechanical stiffness of the cornea through the formation of additional covalent bonds between stromal collagen fibers [1,2]. This is achieved by applying riboflavin (vitamin B2) to the corneal surface and subsequently irradiating it with ultraviolet A (UVA) light [2].

CXL can be performed using two main techniques: the conventional “epithelium-off” method, which requires mechanical debridement of the epithelium to facilitate riboflavin penetration; and the “epithelium-on” (transepithelial) method, which aims to reduce postoperative discomfort and complications, though with potentially lower efficacy due to limited stromal riboflavin absorption [3]. Although epithelium-off CXL is the more commonly used protocol due to its proven long-term effectiveness, it may result in a corneal epithelial defect that requires rapid and complete re-epithelialization to minimize pain, infection risk, scarring, and visual deterioration [4,5].

The speed of corneal epithelial wound healing is a critical component of recovery following CXL. Delayed healing not only prolongs patient discomfort but may also predispose to persistent epithelial defects, stromal haze, and neovascularization [4,5]. Consequently, therapeutic strategies that enhance epithelial repair have in recent years attracted increasing attention [6].

One such strategy is the use of topical insulin. While primarily known for its metabolic role in glucose regulation, insulin also functions as a mitogenic and trophic factor, promoting epithelial cell proliferation, migration, and survival [7]. Insulin and insulin-like growth factor (IGF) receptors are expressed throughout the cornea, and their activation contributes to tissue regeneration and homeostasis, particularly following injury [8]. In preclinical studies, insulin eye drops have been shown to significantly accelerate corneal epithelial healing in diabetic animal models [9], and clinical reports have demonstrated its benefit in patients with neurotrophic keratopathy and persistent epithelial defects [10,11]. A recent review concluded that topical insulin represents a safe, well-tolerated, and cost-effective therapeutic option for promoting corneal surface restoration in select patient populations [12].

In addition to insulin, several other biologically active agents have been examined with the aim of promoting corneal epithelial recovery following injury, including after the epithelium-off phase of corneal collagen cross-linking. Corneal epithelial wound healing is regulated through coordinated activation of the epidermal growth factor receptor (EGFR) pathway and the interplay of multiple growth factors, particularly keratinocyte growth factor (KGF), hepatocyte growth factor (HGF), insulin-like growth factor-1 (IGF-1), and transforming growth factor-β (TGF-β). These signaling networks regulate the proliferation of basal epithelial cells, their migration to resurface the defect, cytoskeletal reorganization, and the re-establishment of epithelial–stromal adhesion via hemidesmosomes. [13]. Any disruption of these processes—whether due to epithelial debridement, oxidative stress from UV A exposure, or postoperative inflammation—may lead to delayed re-epithelialization, prolonged discomfort, stromal haze formation, and slower visual rehabilitation.

Topical recombinant human epidermal growth factor (rhEGF) has been investigated particularly in the management of persistent epithelial defects, where it has demonstrated the ability to enhance epithelial cell proliferation and migration, resulting in quicker defect closure and improved epithelial stability [14]. Another therapeutic strategy focuses on preserving the extracellular microenvironment essential for healing. Heparan-sulfate mimetics (RGTA), such as Cacicol, stabilize endogenous growth factors within the extracellular matrix and protect them from degradation. In a randomized clinical trial in keratoconus patients undergoing standard CXL, RGTA treatment was associated with faster epithelial closure and reduced postoperative pain, highlighting the relevance of matrix preservation in facilitating physiological repair [15].

Neurotrophic support has also gained attention, given the close relationship between epithelial homeostasis and corneal innervation. Recombinant human nerve growth factor (cenegermin) has shown beneficial effects in restoring epithelial integrity and promoting nerve regeneration in neurotrophic keratitis and may be of relevance in situations where corneal sensitivity is transiently reduced following CXL [16]. Similarly, the combined application of substance P and IGF-1 has demonstrated a synergistic effect in enhancing epithelial cell adhesion and migration, further highlighting the role of neuropeptide-mediated signaling in maintaining corneal surface integrity [17].

Within this therapeutic context, topical insulin offers a practical, accessible, and biologically well-founded treatment option. By activating intracellular pathways that overlap with those of IGF-1, insulin supports epithelial cell proliferation and migration and may contribute to more rapid closure of the epithelial defect after CXL. In this case series, we present patients treated with topical insulin drops following epithelium-off CXL and evaluate its effect on the speed of epithelial healing and overall postoperative recovery.

## 2. Materials and Methods

This prospective pilot study included eight patients with progressive keratoconus who underwent standard epithelium-off CXL between January and March 2025 at the Department of Ophthalmology, Clinical Hospital Center Sestre Milosrdnice, Zagreb, Croatia. Before treatment, participants underwent a Schirmer test with anesthetic and TBUT measurements. CLX surgeries were performed in a sterile operating room using the Dresden protocol (0.1% riboflavin + 370 nm UV A at 3 mW/cm^2^ for 30 min). An 8.5 mm area of corneal epithelium was removed in all patients [18]. After the procedure, a corneal bandage contact lens (Air Optix Night & Day^®^; Alcon, Chemin de Blandonnet 8, 1214 Vernier, Geneva, Switzerland) was applied to the operated eye.

Following the procedure, eight patients were prospectively included and assigned into two groups of four to allow for a balanced comparison in this exploratory pilot study. Group allocation was performed using a simple predefined assignment list prepared by a team member. The topical insulin drops were prepared by hospital pharmacy staff under aseptic conditions (the detailed preparation protocol is provided in Appendix A). To minimize potential assessment bias, the surgeon performing the procedure and the examiner measuring epithelial defect size were not informed about group assignment, while administration of the assigned drops (insulin or artificial tears) was performed by a staff member not involved in outcome assessment. The insulin group received topical insulin eye drops (1 IU/mL) five times daily in addition to standard postoperative therapy, which included tobramycin-dexamethasone eye drops two times a day and ofloxacin eye drops 5 times a day. The control group received artificial tears (Systane^®^; Alcon Laboratories, Inc., Fort Worth, TX, USA) five times daily and the same standard therapy.

Corneal epithelial defect areas were quantified using standardized anterior segment photography. Following fluorescein dye application, images were captured at 10× magnification using a slit-lamp mounted digital camera (Topcon; Tokyo, Japan). A single masked observer analyzed all images using ImageJ software (v1.53, National Institutes of Health, Bethesda, MD, USA) to measure defect areas in standardized units (mm^2^). Anterior segment OCT (RTVue, Optovue Inc., Fremont, CA, USA) was performed daily to monitor corneal healing; these images are part of long-term follow-up but are not included in this study and will be reported later.

In addition to imaging, pain intensity (assessed using the Visual Analog Scale, VAS, 0–10) and any adverse effects were recorded.

All patients were monitored daily until complete epithelial healing.

### Statistical Analysis

Descriptive statistics were employed to summarize the characteristics of the patients and their clinical outcomes. Given the small sample size (*n* = 4 per group) and the exploratory nature of this pilot study, all analyses are presented descriptively. Baseline characteristics are shown as raw values (mean ± SD) along with standardized differences (SDiff) to illustrate group balance, without formal hypothesis testing. Postoperative epithelial defect areas and patient-reported visual analog scale (VAS) pain scores were also summarized descriptively using the mean ± SD. Epithelial defects are additionally expressed as a percentage of the theoretical baseline area (56.7 mm^2^) to account for minor measurement variability. Differences between the insulin and control groups are reported as effect sizes with 95% confidence intervals (CIs) to convey the magnitude and direction of treatment effects while acknowledging the uncertainty inherent in such a small sample. Because this is a small exploratory study, we did not perform formal statistical tests for daily comparisons, nor did we adjust for multiple comparisons. Overall, the results should be interpreted as preliminary trends rather than definitive evidence of treatment efficacy. This study adhered to the tenets of the Declaration of Helsinki, and ethical approval was obtained from the Institutional Review Board of the Sestre milosrdnice University Hospital Center (approval number: 003-06/25-03/00, approval date: 14 January 2025.). Written informed consent was obtained from all participants before enrolment in the study.

## 3. Results

### 3.1. Baseline Characteristics

The study included 8 patients (*n* = 4 per group) undergoing corneal collagen crosslinking (CXL) for keratoconus. Baseline demographic and clinical characteristics are presented in Table 1.

### 3.2. Epithelial Defect Size

The mean epithelial defect areas for each group over the three study days are summarized in Table 2. Based on uniform epithelial removal with an 8.5 mm diameter, the theoretical initial defect area was ≈56.7 mm^2^. To account for slight variations in measurements, defect areas were expressed in both absolute terms (mm^2^) and as a percentage of this baseline (“% of theoretical defect”).

On Day 1, the defects were 51.42 mm^2^ (90.7%) in the insulin group and 53.07 mm^2^ (93.6%) in controls, showing minimal difference immediately after epithelial removal. By Day 2, remaining defects corresponded to 13.7% in the insulin group and 20.7% in controls, reflecting faster healing in the insulin-treated eyes. By Day 3, complete epithelial closure was observed in both groups (0%), corresponding to 0% of the baseline defect.

Effect sizes with 95% confidence intervals are provided to illustrate the magnitude and direction of differences between groups, acknowledging the wide uncertainty due to the small sample size (*n* = 4 per group).

**Figure 1:**
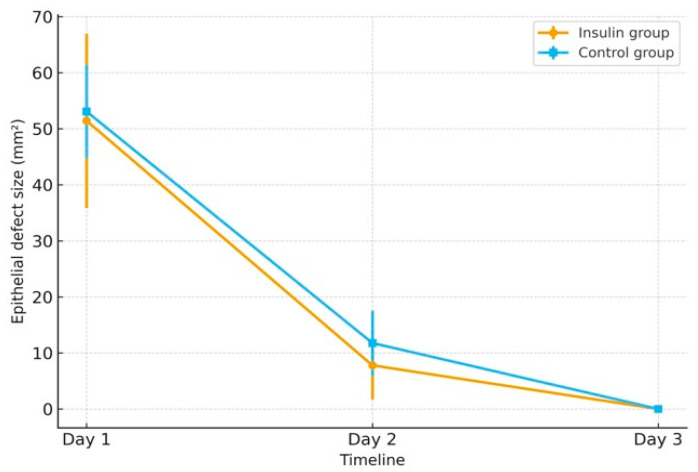
Mean epithelial defect size (mm^2^) from Day 1 to Day 3 in the insulin and control groups. Error bars represent standard deviation.

A line graph (Figure 1) illustrates the decrease in epithelial defect size from Day 1 to Day 3 in both groups, suggesting a faster healing trend in the insulin group; however, this observation was based on a small case series and remains descriptive, warranting confirmation in larger cohorts.

### 3.3. Postoperative Symptoms

Patient-reported pain, assessed using the 0–10 visual analog scale (VAS), was generally low in both groups throughout the postoperative period (Table 2). Due to the small sample size (*n* = 4 per group) and the exploratory nature of the study, VAS scores are presented descriptively as the mean ± SD, and no formal statistical comparisons were performed.

The slight differences in pain scores between groups on Day 1 and Day 2 likely reflect normal inter-individual variability, differences in bandage contact lens comfort, and the subjective nature of pain perception.

### 3.4. Safety

No adverse events (e.g., delayed healing, infection) were observed in either group (Table 2).

**Figure 2:**
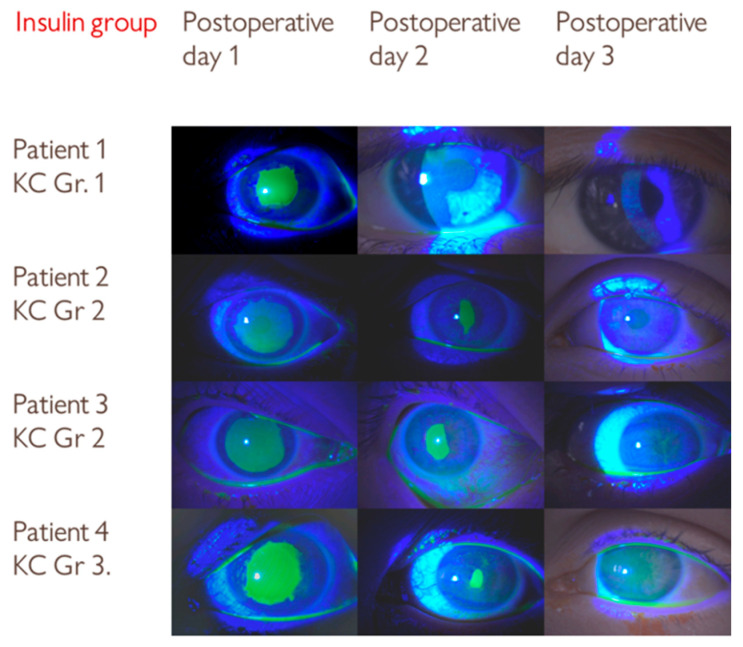
Series of slit-lamp images showing the progression of epithelial defect healing in the insulin group over three postoperative days following corneal collagen crosslinking (CXL) treatment. KC Gr—Keratoconus grade.

**Figure 3:**
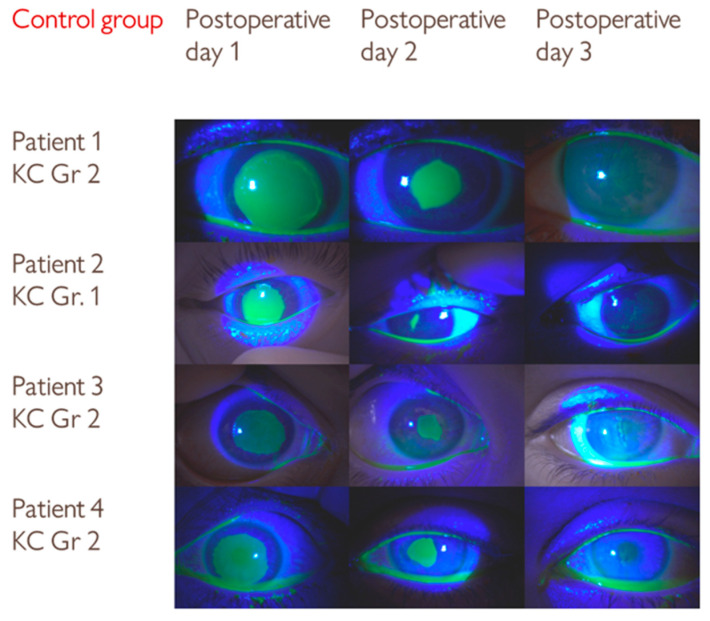
Serial slit-lamp images of an epithelial defect after CXL treatment in the control group. Although the differences in epithelial defect size were not statistically significant, visual comparison with the insulin group suggests a trend toward slightly slower healing.

The distribution of keratoconus stages was similar between the groups (Table 1). In both groups, the patients with grade 1 keratoconus had faster epithelial healing on the second postoperative day (patient 1 in the insulin group and patient 2 in the control group, Figure 2 and Figure 3).

## 4. Discussion

Insulin and its receptors are present in the human tear film and corneal epithelium. Animal and human studies have demonstrated the benefits of the use of insulin drops, such as easy administration, good tolerability, and fewer complications [5]. However, no study to date has evaluated its potential to enhance epithelial healing following corneal collagen crosslinking (CXL).

The corneal epithelium is a self-renewing tissue, maintained by limbal stem cells located at the junction between the cornea and the conjunctiva, which provide new cells that migrate centripetally to replenish the corneal surface. After epithelial debridement, healing begins immediately and follows a complex, coordinated process that includes epithelial cell migration, proliferation, stratification, and re-adhesion to the basement membrane. This process is regulated by numerous cytokines, growth factors (including epidermal growth factor, transforming growth factor beta, and insulin-like growth factor-1), and extracellular matrix components [4].

Despite significant advances in the understanding of corneal epithelial wound healing, effective pharmacological strategies for improving healing are still limited [19]. The standard management of persistent epithelial defects (PEDs) begins with the removal of epithelial-toxic agents and the use of supportive treatments such as preservative-free artificial tears, prophylactic topical antibiotics, bandage contact lenses, punctal occlusion, and topical anti-inflammatory therapy. In more resistant cases, additional options include autologous serum or platelet-rich plasma [11]. If healing still fails, surgical interventions like amniotic membrane transplantation (AMT) may be necessary [7]. The stepwise nature of this therapeutic approach highlights the need for novel pharmacologic agents that can promote epithelial regeneration and prevent complications.

For prolonged corneal epithelial healing or persistent dryness after CXL, treatment involves artificial tears, lubricating ointments, postoperative patch, and antibiotic ointment or bandage contact lens (BCL) [20,21,22]. In refractory cases, autologous serum drops (20–50% concentration) improved healing and reduced postoperative pain versus artificial tears by stimulating epithelial proliferation and migration while delivering concentrated growth factors, cytokines, and vitamins directly to the ocular surface [23]. A 2017 randomized trial [24] found that post-operative application of ReGeneraTing Agent (RGTA) eye drops after epi-off CXL accelerated corneal healing, with 83.3% of eyes fully re-epithelialized by Day 2 (vs. 13.3% in controls) while also reducing pain, photophobia, and irritation symptoms. So far, recombinant human epidermal growth factor (rhEGF) showed mixed results in clinical trials [25,26] while amniotic membrane remains an effective option for persistent epithelial defects and for preventing sterile corneal melting after CXL [27].

In recent years, topical insulin has emerged as a promising adjunct therapy to support epithelial healing. Preclinical studies suggest that insulin enhances healing by activating the PI3K/Akt and MAPK pathways and accelerating wound closure in rodent models [28]. In diabetic mice, insulin has been shown to promote both epithelial wound closure and corneal reinnervation, likely via Wnt/β-catenin signaling modulation [29].

Clinical studies in diabetic keratopathy have demonstrated the efficacy of insulin. In one study, insulin at 1 IU/mL applied four times daily led to significantly faster epithelial healing compared to artificial tears, reducing the healing time by 54% [28,30]. In the treatment of recurrent corneal erosions, topical insulin combined with standard therapy resulted in no recurrence during follow-up, while the control group had a recurrence rate of 21.4% [31]. Moreover, topical insulin led to complete epithelial closure within one week in a case of bilateral neurotrophic keratitis refractory to other treatments (25 IU/mL, six times daily) [32].

Most studies investigating topical insulin targeted chronic or complex corneal defects rather than the controlled epithelial injury induced by CXL, highlighting a gap this study addresses by evaluating insulin—a molecule with proven mitogenic effects in other ocular surface pathologies.

This study presents a case series of 8 patients with progressive keratoconus who, following CXL, received either topical insulin (1 IU/mL, dosage and mixture consistent with prior studies [7]) or artificial tears as an adjunct therapy to standard treatment. Daily monitoring continued until corneal epithelium fully regenerated, enabling a direct comparison of healing speed between the two therapeutic approaches.

On the first postoperative day, the insulin group displayed greater variability in epithelial defect size (reflecting a larger standard deviation), despite identical epithelial removal diameter in all patients. This variation likely reflects natural differences in individual epithelial healing dynamics. By the second postoperative day, the difference between groups became smaller, with a trend toward smaller remaining defects in the insulin group (Table 2), consistent with the known proliferative and wound-modulating effects of insulin. By the third postoperative day, complete epithelial closure was observed in both groups.

Compared with studies investigating larger or persistent epithelial defects, the trend toward faster epithelial closure observed with topical insulin in our cohort appears more modest. This difference may reflect distinct pathophysiological contexts: CXL produces a controlled, self-limiting epithelial injury with intrinsically rapid healing potential, whereas insulin has shown greater clinical benefit in chronic or non-healing corneal surface disease. The UV–riboflavin process induces cytokine-driven epithelial regeneration (including IL-6 and TGF-β), and the transient post-CXL reduction in sub-basal nerve density typically recovers without prolonged epithelial compromise. However, in selected high-risk patients—such as those with neurotrophic dysfunction, diabetes mellitus, delayed healing beyond 72 h, persistent epithelial defects, or ocular surface instability—topical insulin may offer clinically meaningful benefit and warrants further investigation in larger controlled cohorts [2].

Previous clinical trials showed mixed results when comparing insulin eye drops to artificial tears: while equivalent to artificial tears for diabetic DED symptoms [33], it outperformed them in healing post-vitrectomy defects [30] and preventing erosion recurrence [31].

Postoperative pain, assessed using the Visual Analog Scale (VAS), showed a gradual decrease over the first three days in both treatment groups. Despite slight numerical differences, statistical analysis did not reveal any significant advantage of insulin drops in terms of pain reduction. These findings align with previous reports suggesting that postoperative discomfort after CXL is multifactorial and not significantly altered by adjunctive topical treatments [34,35,36].

In keratoconus patients, stage-dependent differences in corneal healing capacity exist: Stage I (>500 μm) demonstrates preserved stromal reserves, organized epithelium, and normal tear film [37,38] while Stage II (400–500 μm) shows epithelial instability (microcysts, reduced hemidesmosomes), higher risk of deeper stromal damage during UV application, tear film dysfunction (lower TBUT), and amplified inflammatory response (elevated IL-6/TNF-α, increased keratocyte apoptosis and metalloproteinase (MMP-9) activation) [39,40,41] with slower healing rates, particularly below 400 μm [42].

In our study, faster epithelialization in Stage I can be explained by the preserved corneal thickness, better epithelial organization, and a lower inflammatory response. This highlights the importance of classifying participants by stages of keratoconus in future studies.

This study has several limitations. The small sample size (*n* = 8) restricts the ability to draw firm statistical conclusions, and the fixed insulin dosing regimen may not reflect the optimal therapeutic range due to potentially limited corneal penetration. Additionally, the study focused on early epithelial healing up to Day 3 and does not report the refractive outcomes, long-term haze, or other delayed complications. Although these outcomes were monitored during follow-up, they were beyond the scope of the present analysis and will be addressed in future reports. Further investigations are warranted, including adequately powered randomized controlled trials, objective and standardized methods for wound assessment, and dose-ranging studies, to more precisely define the therapeutic potential of insulin in corneal epithelial healing following CXL.

Beyond these limitations, recent literature has increasingly explored the broader biological rationale for using insulin in ocular surface repair. Insulin and IGF-1 signaling are now understood to play a key role not only in epithelial proliferation, but also in modulating the extracellular matrix and epithelial–stromal adhesion complexes, which are essential for stable resurfacing following injury [8,9]. The integrity of hemidesmosomes and anchoring fibrils is particularly relevant in the early post-CXL period, when the epithelial basement membrane undergoes remodeling in response to UV-A–induced cross-linking effects. If re-adhesion is incomplete or delayed, patients are at increased risk of recurrent erosions, postoperative pain, delayed visual rehabilitation, and stromal haze development [6]. By promoting cytoskeletal organization and supporting re-anchoring mechanisms, topical insulin may theoretically reduce these risks, although this has not yet been demonstrated in controlled studies.

Recent clinical reports have also emphasized the safety profile and feasibility of compounded insulin formulations for ophthalmic use. Stability studies confirm that properly prepared dilutions maintain biological activity and sterility over clinically relevant storage intervals [7], and multiple case series have documented good tolerability without significant local or systemic adverse events, even with prolonged use in refractory persistent epithelial defects [5,11,12]. This is an important consideration for CXL patients, among whom epithelial healing responses can vary widely depending on disease stage, ocular surface quality, and systemic factors such as diabetes mellitus.

The options for supporting corneal epithelial regeneration are still evolving, with new pharmacologic and biologic approaches being explored to complement traditional supportive care. While autologous serum, platelet derivatives, RGTA matrices, nerve growth factor analogs, and amniotic membrane transplantation each provide targeted benefits, they also present limitations related to cost, accessibility, regulatory approval, donor variability, or the need for surgical intervention. In contrast, insulin is inexpensive, widely available, and familiar to clinicians, positioning it as a potentially valuable adjunct therapy in selected patient groups. However, its optimal dosing, timing, and duration in the context of CXL remain undefined. The present findings, although preliminary, support the concept that insulin may provide measurable benefit, particularly in patients predisposed to delayed epithelial healing—such as those with advanced keratoconus, ocular surface inflammation, diabetes, or neurotrophic compromise.

Future studies should therefore aim to stratify patients by keratoconus severity, ocular surface status, and systemic comorbidities to better determine which subgroups may derive the greatest benefit. Incorporating objective epithelial imaging tools (e.g., anterior segment OCT, in vivo confocal microscopy) and standardized pain assessment metrics would further improve data quality. Ultimately, well-designed randomized controlled trials will be necessary to confirm whether insulin can meaningfully accelerate healing or reduce complication rates in the early postoperative period following CXL.

## 5. Conclusions

This study provides the first clinical insight into the potential role of topical insulin in supporting corneal epithelial healing after epithelium-off CXL. Although based on a small number of patients, the findings suggest that insulin is well-tolerated and may modestly speed up early epithelial closure. However, the observed treatment effect appears modest, and the small cohort limits the applicability of the results. These preliminary observations support further research in larger controlled trials to determine the best dosing, treatment duration and the possible benefits of combining insulin with other regenerative therapies, as well as evaluate long-term outcomes such as visual recovery, corneal clarity, and the incidence of postoperative complications.

## Figures and Tables

**Table 1:** Baseline demographic and clinical characteristics.

Parameter	Insulin Group (*n* = 4)	Control Group (*n* = 4)	Standardized Difference (SDiff)
Mean age (years)	29.8 ± 4.8	29.8 ± 4.8	0.48
Sex (M/F)	2/2	3/1	-
Keratoconus stage	2.00 ± 0.82	1.75 ± 0.50	0.34
Preop. Central corneal thickness (CCT) (μm)	489.00 ± 39.56	504.00 ± 22.82	0.44
Schirmer test (mm)	21.0 ± 10.15	17.5 ± 13.08	0.29
TBUT (sec)	8.0 ± 2.00	10.0 ± 4.40	0.58

Data are presented as the mean ± standard deviation unless otherwise indicated. Standardized differences (SDiff) represent the absolute difference in means divided by the pooled standard deviation, providing a measure of baseline balance between groups. No statistical tests were performed due to the small sample size (*n* = 4 per group) and exploratory design of the study.

**Table 2:** Daily postoperative epithelial defect size, percentage of theoretical defect, effect size versus control (95% CI), visual analog scale (VAS) pain scores, and adverse events in the insulin and control groups.

Timeline	Group	Epithelial Defect (Mean ± SD) (mm^2^)	% of Theoretical Defect (Mean ± SD)	*n*	Effect Size vs. Control (95% CI)	VAS (0−10) (Mean ± SD)	Side Effects
Day 1	Insulin	51.42 ± 15.56	90.7 ± 27.5%	4	−1.65 (−15.0 to +11.7)	4.5 ± 1.0	No
Day 1	Control	53.07 ± 8.33	93.6 ± 14.7%	4	Reference	2.5 ± 3.3	No
Day 2	Insulin	7.79 ± 6.07	13.7 ± 10.7%	4	−3.98 (−14.0 to +6.0)	2.0 ± 2.3	No
Day 2	Control	11.77 ± 5.81	20.7 ± 10.3%	4	Reference	2.5 ± 2.0	No
Day 3	Insulin	0	0%	4	0	1.0 ± 1.4	No
Day 3	Control	0	0%	4	Reference	0.5 ± 1.0	No

Epithelial defect areas after the uniform removal of an 8.5 mm diameter corneal epithelium. “% of theoretical defect” represents the measured defect as a percentage of the theoretical maximum area (56.7 mm^2^). “Effect size vs. Control (95% CI)” indicates the mean difference between insulin and control groups with 95% confidence intervals; *n* = 4 per group. VAS (0–10) shows patient-reported pain scores. Side effects were monitored daily.

## Data Availability

The data presented in this study are available on request from the corresponding author. The data are not publicly available due to privacy and ethical restrictions.

## Abbreviations

The following abbreviations are used in this manuscript:

CXL	Corneal cross-linking
AS-OCT	Anterior Segment Optical Coherence Tomography
UVA	Ultraviolet-A
IGF	Insulin-like growth factor
TBUT	Tear break-up time
VAS	Visual Analog Scale
SD	Standard deviations
CCT	Central corneal thickness
PEDs	Persistent epithelial defects
AMT	Amniotic membrane transplantation
BCL	Bandage contact lens
RGTA	ReGeneraTing Agent
rhEGF	Recombinant human epidermal growth factor
DED	Dry eye disease
MMP-9	Metalloproteinase

## Appendix A. Detailed Preparation of Topical Insulin Drops

Due to the absence of commercial insulin eye drops for corneal healing, topical insulin formulations were prepared in our hospital pharmacy under aseptic conditions using licensed injectable insulin. Preparation was based on a previously published protocol [7], with adaptations:

- Insulin used: Humulin R 100 IU/mL (Eli Lilly and Company, Indianapolis, IN, USA).
- Vehicle: Systane Ultra^®^ artificial tears.
- Final concentration: 1 IU/mL in a total volume of 10 mL.
- Preparation: 0.1 mL of Humulin R was mixed with 9.9 mL of Systane Ultra under a laminar airflow hood.
- Storage: Drops were considered usable for up to 7 days if refrigerated and protected from light.
- Note: No independent concentration verification or stability testing was performed.
